# Study of childhood onset schizophrenia (COS) using SPECT and neuropsychological assessment

**DOI:** 10.4103/0019-5545.31552

**Published:** 2006

**Authors:** Savita Malhotra, Nitin Gupta, Anish Bhattacharya, Mehak Kapoor

**Affiliations:** * Professor, Department of Psychiatry, Post Graduate Institute of Medical Education and Research (PGIMER), Chandigarh; ** Professor, Department of Psychiatry, Post Graduate Institute of Medical Education and Research (PGIMER), Chandigarh; **** Assistant Professor, Department of Psychiatry, Post Graduate Institute of Medical Education and Research (PGIMER), Chandigarh; *** Professor, Department of Nuclear Medicine, PGIMER

**Keywords:** Childhood onset schizophrenia, SPECT, schizophrenia

## Abstract

**Background:**

In recent years, the development of positron emission tomography (PET) and single-photon emission computed tomography (SPECT) imaging has enhanced our understanding of the physiological functioning of the intact brain.

**Aim:**

To study cerebral cortical perfusion defects in patients with childhood onset schizophrenia (COS) and to assess their neuropsychological functioning.

**Methods:**

This cross-sectional study comprised 14 patients with COS with onset at or before 14 years of age, diagnosed as per ICD-10 DCR criteria, attending a tertiary care centre in North India. All the patients were assessed on sociodemographic, clinical profile sheet, Positive and Negative Symptom Scale (PANSS) and Edinburgh Handedness Inventory (EHI). The Wisconsin Card Sorting Test (WCST) was used to assess their neuropsychological functioning. All patients underwent SPECT. A control group of 10 healthy subjects was studied with SPECT for comparison.

**Results:**

Nine patients (64.3%) showed perfusion anomaly on SPECT scan specifically in the left temporal and frontal areas of the brain. On WCST score these 9 patients showed a higher percentage of total errors (64.49%±9.42%) as compared to the other 5 patients (48.54%±12.70%) who showed no abnormality on SPECT scan. All normal control subjects showed no abnormality on SPECT.

**Conclusion:**

The results from WCST show that COS patients have difficulty in executive functioning. Also, patients had perfusion anomaly in the left temporal, frontal and parietal areas. Deficits found in COS are similar to those found in adult onset schizophrenia (AOS). In view of the findings, the nature of COS and its relationship with AOS are discussed.

## INTRODUCTION

Schizophrenia is most commonly seen during early adulthood^1^,^2^ but can manifest itself in extreme of ages, i.e. in childhood^3^–^7^ and elderly population.^8^,^9^ Childhood onset schizophrenia (COS) occurs in early childhood (up to puberty). There is controversy regarding the cut-off age used for diagnosis of COS. However, the most acceptable cut-off age is taken to be < 14 years.^5^,^7^,^10^,^11^ It is a very rare form of schizophrenia and is associated with greater disease severity and heritability than adult onset schizophrenia (AOS).^12^ Remschmidt *et al.*^7^ reported that 0.1%–1% of all schizophrenic disorders manifest themselves before 10 years of age and 4% before 15 years of age. In both DSM-IV^13^ and ICD-10,^14^ COS is diagnosed using criteria for adult schizophrenia and is largely phenomenological.

In recent years, the development of positron emission tomography (PET) and single-photon emission computed tomography (SPECT) imaging has increased our understanding of the physiological functioning of the intact brain. SPECT allows assessment of brain metabolic activity by measuring cerebral blood flow.^15^,^16^ Such progress has allowed us to use diagnosis in more clinically relevant ways, given us important pharmacological treatments and enhanced our understanding of the potential relationship between brain structures, static measures of blood flow and a variety of neurological and psychiatric disorders. SPECT has major strengths for clinical use in neuropsychiatry.^17^,^18^ The first is its capacity to provide three-dimensional measurements of regional cerebral blood flow long after the injection of the radiotracer. The second is its availability and affordability.^19^ SPECT has found several clinical applications in the field of psychiatry. Functional brain imaging methods have been applied to study schizophrenia that aim at elucidating the neurobiology of this complex and heterogeneous disorder.^20^

There are three groups of findings on functional neuro-imaging in schizophrenia:

- Hypofrontality
- Altered basal ganglia perfusion and metabolism
- Altered temporal lobe perfusion and metabolism.

The major finding in schizophrenia is ‘hypofrontality’, seen in the frontal cortex. Many studies have compared schizo-phrenia patients with healthy volunteers using either PET or SPECT imaging, or the non-two-dimensional non-tomographic blood flow method. The majority of these studies show hypofrontality in patients with schizophrenia.^21^–^23^ Schizo-phrenic patients show increased ventricular size with decreased brain volume when compared to non-schizophrenic controls.^24^ In the area of basal ganglia the data on perfusion or metabolic abnormalities in schizophrenia are conflicting. A study on 10 patients with first acute schizophrenia and controls using TRODAT-1 ([^99m^Tc] TRODAT-1) revealed that mean specific TRODAT-1 binding in the striatum did not differ significantly between the patient and age- and sex-matched control group (1.25 versus 1.28). Variance was significantly higher in the patient group.^25^

In COS, non-invasive methods for studying brain structure and function have lead to a small body of research.^26^ COS is rare and severe form of schizophrenia and offers an important opportunity to examine abnormal neurodevelopment in this disorder. Studies of this population have demonstrated clinical continuity with AOS,^27^,^28^ along with more severe premorbid language impairments^29^ and a more chronic course,^10^ possibly reflecting a more extensive developmental brain lesion. Quantitative brain magnetic resonance imaging (MRI) of patients of COS in the National Institute of Mental Health (NIMH) study revealed smaller cerebral volume, significantly smaller than normal mid-sagittal thalamic area, increased basal ganglia, larger than normal lateral ventricular volume, which appears to be progressive, and no abnormality in the size of medial temporal lobe structures.^30^–^32^ Smaller volumes of cerebellar vermis^33^ and four-fold greater decrease in cortical grey matter volume during adolescence with a disease specific reduction in frontal and temporal regions^34^ has seen reported in patients with COS. Abnormal cerebellar blood flow and metabolism have also been demonstrated in COS.^33^

Reports are scanty regarding perfusion studies in COS mainly because of scarcity of patients. Technical difficulties of doing cerebral SPECT on these patients and operationalizing criteria for disorders, in which key symptoms are aspects of subjective experience, are inherently difficult. This is further compounded by developmental limitations of children in describing complex internal symptoms and overlap between psychopathological phenomena and normal experience of childhood (e.g. vivid fantasy versus delusion).^35^ It is known that earlier the onset of schizophrenia, more are the biological factors involved. Hence COS is more of a ‘biological’ disorder.^36^,^37^ The functional assessment of PET and SPECT are new tools of neuroimaging that would give a better understanding of the biological basis of schizophrenia in childhood by determining the decreased blood perfusion in various areas of the brain. This would provide crucial information about pathophysiology and basic cognitive neuroscience. SPECT has greater advantage in studying conditions which may require sedating the patient for scanning as the radiotracer gets deposited in active brain regions within 2–5 minutes of injection and the perfusion patterns are already fixed and can be picked up long after the injection.

### Aim

To study cerebral cortical perfusion defects using SPECT and neuropsychological functions in patients with childhood onset schizophrenia.

### Objectives

1. To do a SPECT study on patients diagnosed as COS and compare it with normal brain SPECT findings.
2. To study neuropsychological correlates of perfusion abnormalities seen in COS patients.

## METHODS

### Sample

A cohort of patients diagnosed as COS (with onset at ≤ 14 years of age) undergoing treatment at Child and Adolescent Psychiatry Clinic of PGIMER, Chandigarh, over one year were taken as study subjects.

### Inclusion Criteria

1. Diagnosis of schizophrenia as per ICD-10.
2. Patients should have stable psychopathology which was defined as a condition where patients did not exhibit violence, aggression or did not reach to psychotic symptoms.
3. Patients and families cooperated for the study and gave consent.

### Exclusion Criteria

Patients with comorbid conditions, i.e. substance abuse, organic brain disorder including epilepsy and movement disorder.

A total of 14 subjects (7 girls, 7 boys) with COS were included in this study. The group as a whole had undergone substantial treatment in terms of neuroleptic therapy (mean exposure 5.08 years, SD 4.33).

A control group of 10 subjects who had no physical or mental illness and who were a part of research data in the Department of Nuclear Medicine of the Institute was taken as controls.

### Assessments tools

- Sociodemographic characteristics.
- Clinical profile of patients that included age of onset, duration of illness, ICD-10 subtype diagnosis, type of treatment and treatment response.
- Severity of psychopathology was rated on the positive and negative symptom scale (PANSS). This has 30 items grouped into 3 areas—positive scale (7 items), negative scale (7 items) and general psychopathology scale (16 items). Each item is rated on a 7-point scale. Total composite index is obtained; higher score indicates more psycho-pathology.
- Handedness: The Edinburgh Handedness Inventory (EHI) is a 10-item self-rated questionnaire. The ratings are dichotomous. The scale is designed for quantitative assessment of handedness. It calls for answers to questions about the subject's practice in performing a number of habitual everyday acts in which the roles of right and left hands are supposed to be sharply distinguished. The inventory provides a standard of comparison in neuro-psychological work.^38^
- Neuropsychological assessment was done using the Wisconsin Card Sorting Test (WCST), which is a measure of the executive functioning. The WCST consists of four stimulus cards and 128 response cards depicting figures of varying forms, colours and numbers of figures. The four stimulus cards are placed before the subject in a particular order. The subject is then handed a deck of 64 response cards and instructed to match each consecutive card from the deck with one of the four stimulus cards. The subject is told only whether each response is right or wrong and is never told the correct sorting principle which keeps changing after specific number of consecutive ‘correct’ responses.^39^
- SPECT Image acquisition: The tracer (^99m^sub Tc-ECD dose 20 mli, 740 mBq) was administered intravenously in a quiet room with dimmed lights 40 minutes prior to the SPECT data acquisition. The patient's head was positioned along the orbitomeatal line perpendicular to the floor in a head holder with an adjustable angle along the laterolateral axis. Head motion was minimized, if required by securing it in position with velcro chin and head straps. Imaging was performed on a dual-head gamma camera (E Cam, Siemens, Germany) fitted with low-energy high-resolution (LEHR) collimators, using the standard brain SPECT acquisition protocol at our institute. Sixty-four planar images were acquired (32 images per camera head) at 40 seconds per image during a 360° rotation around the patient's head. The raw data thus acquired was transferred to an online computer (Integra, GE, USA) and processed to obtain transverse, sagittal and coronal sections of the brain (image).^40^ Two slices were analysed: one 4 cm and the other 6 cm above the orbitomeatal line. The regions of interest (ROIs) were analysed using a semi-automatic brain quantification program. The slices were first rotated and re-aligned so that the transaxial, sagittal and coronal slices were at 90° to each other. Then, multiple round or oblong regional ROIs were drawn on the right side of the brain and mirrored on the left side by hand by an experienced observer blind to the subject's diagnostic status. ROIs were defined individually over different cortical areas based on the system of Talairach *et al.*^41^ This system has also been used by Tanaka *et al.*^42^ to determine ECD SPECT patterns in healthy adults. The average counts per pixel in each ROI was determined and the ratio of these counts to the ipsilateral cerebellar ROIs counts calculated. The cerebellum was used for normalization since the reliability of this method has been examined in patient and normal controls.^43^ The average repetition error (or precision) across regions of interest was about 10%.

### Consent

Written informed consent was obtained from patients and caregivers at the start of the study. Informed consent included consent to participate in the physical assessment; right to refusal or withdrawal of consent; confidentiality of information; possible risks and benefits of the procedure. For the administration of rest of the instruments verbal informed consent was taken from all the patients and caregivers. Similar consent was taken from the subjects in the control group. The study was approved by the Ethics Committee of the Institute.

### Procedure

Patients meeting the selection criteria were taken from the psychiatry outpatients follow-up clinic during one year. Patients and their families were contacted through letters, followed by telephone calls with the request to visit the psychiatry OPD along with the patient on a specified date for further evaluation and specialized investigation. Assessments on all the scales, viz. sociodemographic profile, clinical profile, PANSS and handedness were carried out. SPECT was done for all the patients. Neuropsychological assessment (WCST) was carried out before the SPECT study on the same day. All assessments were cross-sectional.

### SPECT Analysis

The eye balling technique was used to assess the reduced tracer uptake. Ratios of pixel counts of the ROIs to the cerebellum of the corresponding side were calculated. Finally a comparison of ratios on the left was done with the corresponding area on the other side and a difference of more than 10% was taken to be significantly reduced perfusion.

Descriptive analysis was used for sociodemographic and clinical variables and to compare WCST and PANSS score, Mann–Whitney test was used.

### Results

Table 1 shows the demographic profile of both the patient as well as the control groups. Table 2 shows the clinical profile of the COS group. The mean age of onset was 12.4 years (±1.37) and the mean total duration of illness at assessment was 7.45 years (±4.52). Seven (50%) patients had undifferentiated schizophrenia and 9 (64%) were on atypical antipsychotics.

**Table 1:** Sociodemographic variables

Variables	COS (%) (*n*=14)	Normal controls (%) (*n*=10)
Age (years)	20.14	27.00
Mean (SD)	(4.42)	(1.05)
*Sex*		
Male	7(50)	8 (80)
Female	7(50)	2(20)
*Marital status*		
Single/others	14 (100)	6 (60)
Married	0	4 (40)
*Education*		
Matric or below	7 (50)	0
Above matric	7(50)	10 (100)
*Family type*		
Nuclear	10 (71.4)	8 (80)
Others	4 (28.6)	2(20)
*Locality*		
Urban	13 (92.9)	10 (100)
Rural	1 (7.1)	0


*In the control group all the subjects were employed and in COS group only 2 were employed and most others were students.

**Table 2:** Clinical variables

Variable	COS (n=14)
Age at onset (years)	
Mean (SD)	12.4 (1.37)
Total duration of illness (years) at assessment	
Mean (SD)	7.45(4.52)
ICD-10 schizophrenia subtype	
Undifferentiated	7
Catatonic	2
Paranoid	4
Unspecified	1
Type of antipsychotic	
Typical	3
Atypical	9
Both	2
Response to medicine	
Good	7
Poor/partial	7
Side-effects	
Yes	7
No	7

Table 3 shows the perfusion status on SPECT. It was seen that 5 patients had normal perfusion and rest of the 9 had reduced perfusion in different areas of the brain. All the patients were right handed.

**Table 3:** Perfusion status on SPECT

Subjects	Reduced perfusion*
1	Normal perfusion
2	Left fronto-temporal*
3**	Left temporal*
4	Normal perfusion
5***	Left frontal*
6	Right thalamus*
7	Left temporo-parietal*
8	Left caudate nucleus*
9	Normal perfusion
10	Normal perfusion
11	Right thalamus temporal*
12	Right temporo-parietal*
13	Left fronto-temporal*
14****	Normal perfusion

* All the patients were right handed

*** Fig. 3

** Fig. 2

**** Fig. 1

Table 4 shows that the hypoperfusion was mostly localized in the left side as compared to the right and lateralization was maximum in the temporal lobe followed by the frontal lobe.

**Table 4:** Localization and lateralization of perfusion defects

	Area	Number
1.	Frontal lobe	3
2.	Parietal lobe	2
3.	Temporal lobe	6
4.	Right thalamus	2
6.	Caudate nucleus	1

Right versus left 3:6

Table 5 shows the PANSS and WCST scores for both the groups were calculated and were compared using Mann–Whitney test. There were no significant differences found on PANSS score on both the groups. For WCST the percentage total error was significantly higher in the hypoperfusion group as compared to the normal group as shown in table.

**Table 5:** PANSS and WCST score for the two groups

	Group 1 Normal perfusion (*n*=05)		Group 2 Hypoperfusion (*n*=9)	
**PANSS (Mean, SD)**				
Positive scale	14.20	(7.79)	14.89	(8.94)
Negative scale	24.0	(13.26)	20.67	(9.58)
General psychopathology	33.6	(18.07)	34.00	(12.63)
**WCST (Mean, SD) (in**%)				
Total error	48.54	(12.70)	64.49*	(9.42)
Perseverative error	36.18	(16.98)	47.39	(21.41)
Non-perseverative error	16.51	(3.98)	17.09	(14.54)
Perseverative responses	38.96	(19.48)	62.45	(27.86)

* Mann-Whitney u=6.5; p<0.05

Table 6 shows the WCST scores of patients with hypo-perfusion in frontal lobe which was twice as common as frontal lobe and scores of patients with hypoperfusion in other areas. Perseverative responses were higher in the group with hypoperfusion in frontal area. No statistical analysis was used on these two groups, as the sample size was very small.

**Table 6:** WCST scores in patients with frontal-lobe versus other areas of hypoperfusion

	Frontal lobe hypoperfusion (*n*=3) (Mean, SD)		Hypoperfusion in other areas (*n*=6) (Mean, SD)	
Total number of errors	62.76	(14.41)	65.36	(7.50)
Perseverative errors	46.09	(29.49)	48.04	(19.59)
Non-perseverative errors	16.66	(15.24)	17.31	(15.66)
Perseverative responses	68.1	(27.95)	59.63	(30.01)

### Discussion

Findings in our study revealed hypoperfusion mostly in the temporal lobe; which was twice as common as frontal lobe, in the frontoparietal region and also in subcortical areas in COS subjects whereas normal controls had no perfusion defects. Lateralization for right versus left was 3:6 indicating left-sided impairment.

The current understanding of schizophrenia as a neuro-developmental disorder is largely due to brain imaging studies.^44^–^46^ SPECT has helped to elucidate the neurobiology of schizophrenia via the study of cerebral blood flow and neuroreceptors in this condition. There is converging evidence implicating three brain systems—frontal, temporoloimbic and basal ganglia.^47^

In our study the perfusion defects were mostly localized in the temporal lobe (43%). Functional neuroimaging studies, utilizing PET and SPECT have consistently reported temporal lobe perfusion abnormalities in individuals with schizo-phrenia.^48^–^50^ Zakzanis, *et al.*^51^ in their meta-analytic study of structural of structural (CT and MRI) and functional (SPECT and PET) studies propose the major involvement of temporal system in schizophrenia. A study trying to see the brain activation during the passive viewing of emotional and neutral facial expressions in schizophrenia using fMRI showed that relative to control subjects, patients demonstrated elevated hippocampal and amygdala activation during the passive viewing of human faces.^52^

Results from our study revealed that the frontal lobe (21%) was the second most common lobe with perfusion defects followed by parietal lobe (14.3%), right thalamus (14.3%) and caudate nucleus (7.4%). A substantial number of studies have shown frontal lobe dysfunction and dysfunction in subcortical areas. In a study on active and remitted schizophrenics it was found that during the active phase of illness, cerebral perfusion patterns in schizophrenic patients was characterized by both hypofrontality and hypotemporality and after remission hypofrontality was no longer apparent in two of four frontal regions, and hypotemporality disappeared completely.^53^ In a study on five subjects with simple schizophrenia it was found that three of the five simple schizophrenia patients had findings of atrophy and reduced cerebral perfusion in the frontal areas. Voxel-based analyses also showed pre-frontal grey matter deficits and hypoperfusion in simple schizophrenia patients compared with the controls.^54^ In another study which reviewed all studies published in English on schizophrenia using PET, SPECT, xenon studies, functional and spectroscopic MRI, the most consistent finding was that of hypofrontality.^55^ In a study by Li *et al.*^56^ on 45 schizophrenic patients, it was found that schizophrenic patients showed hypoperfusion in the frontal and temporal lobes and hyperperfusion in the basal ganglia. More than 20 studies in patients with schizophrenia were reviewed and results showed that patients are more likely to be low in glucose uptake in areas of frontal lobes, basal ganglia and temporal lobe than in occipital lobe, cerebellum, or in white matter.^21^ A substantial number of patients with schizophrenia exhibit primarily frontal and temporal lobe dysfunction pointing towards neurobiological heterogeneity in schizophrenia.^57^ Hypofrontality has emerged as the strongest finding in MRI, PET studies in schizophrenia.^22^ However, there is also evidence of hyperfrontality available. Manoach^58^ in his study stated that both hypo- and hyperfrontality are hypothesized to be valid and informative reflections of prefrontal cortex dysfunction in schizophrenia.

Results in our study showed more left side impairment as compared to right with six impairments on the left side and three on the right. In a study which examined temporal lobe perfusion using ^99m^technitium hexamethyl propylene amine oxine (HMPAO)-SPECT in schizophrenic patients, results showed significant left hypoperfusion relative to right of their temporal lobes.^59^ Gur *et al.*^60^ highlighted the application of brain imaging in schizophrenia research emphasizing on studies that evaluated hemispheric differences. There is evidence for lateralized abnormalities in some studies and in general, the results implicate abnormalities in left hemispheric activity.

Our findings differed from a study of quantitative brain MRI of COS patients reported from the National Institute of Mental Health (NIMH) USA, which revealed no abnormality in the size of medial temporal lobe structures, significantly smaller than normal mid-sagittal thalamic area and larger than normal lateral ventricular volume.^10^,^30^–^32^ This can be due to several reasons such as difference between structural (MRI) and functional assessments (SPECT), sensitivity and specificity of investigations, difference in the patient population under study, not using drug naive patients.

It was found that the two groups, viz. hypoperfusion and normal perfusion did not differ on clinical psychopathology. Previous research has shown that positive and negative symptoms of schizophrenia are related to dysfunction in different regions of the brain and different lateralized patterns of dysfunction.^61^

According to the available literature the most common finding in SPECT functional imaging is a reduction in frontal blood flow and subsequent poor performance on suspected frontal lobe tests.^62^ In our study, patients with hypoperfusion showed significantly higher percentage of total error and perseveration was also higher in this group. However, within the hypoperfusion group, perseveration was higher in the group with hypoperfusion in the frontal areas as compared to that in other areas. The results from WCST show that COS patients have problems in executive functioning which is a frontal lobe function. Also, patients had perfusion anomaly in the left temporal as well as in the parietal, thalamic and basal ganglia region. Thus this study suggests that deficits found in COS could be due to temporo-fronto-parietal localization of pathology in the brain. Definite causal relationship between hypoperfusion and clinical manifestations of schizophrenia cannot be positively established in this study. Whereas the executive functions are largely subserved by the frontal cortex, they are also related to other parts of the brain that have strong connections to the frontal cortex such as the temporal limbic complex.

In our study, COS subjects showed very high frequency of temporo-frontal-subcortical (thalamus and caudate nucleus) hypoperfusion on SPECT which was also associated with higher perseverative errors. The left brain was more often involved as compared to the right.

In schizophrenia, there are patterns of hypofrontality coupled with an increase in metabolism in subcortical regions particularly the basal ganglia.^21^ Studies indicate that COS patients are more severely affected than AOS as the total brain volume in COS is 9.2% lesser compared to 3% lesser in AOS; there is progressive increase in ventricular brain ratio; 17% more reduction in midline thalamus volume in COS.^30^ As such, findings in COS are similar to those in AOS. This implies that COS falls on a continuum with AOS. However, greater frequency of temporal lobe involvement in COS patients towards brain insult occurring very early in brain development leads to not only a very early onset of illness but also a much greater severity of schizophrenia in COS. As the frontal lobe continued to mature post-nataly until adolescence, any abnormalities in neurodevelopment during this phase can manifest as with frontal lobe dysfunctions as well as symptoms of schizophrenia. Studies comparing COS, adolescent onset and adults should be done using SPECT which are likely to further highlight the neurodevelopmental hypothesis and also the possible differential lober involvement in schizophrenia.

There is also need to correlate SPECT findings with clinical disease variable such as type of onset, duration of illness and nature of psychopathology in larger samples in order to arrive at clear correlates of underlying brain pathology.

## CONCLUSION

Schizophrenia has been considered to be a neurodevelopmental disorder and the COS offers an important opportunity to examine abnormal neurodevelopment in this disorder. SPECT and neuropsychological findings in COS converge with those of AOS but with greater temporal lobe involvement in COS. This is the first SPECT study in COS; therefore, the findings could be compared only with MRI studies that are available and depicts structural abnormalities. There is complex interaction between structural and functional systems in the CNS. As SPECT studies advance it may be possible to physiologically characterize, classify and diagnose various neurobiological and psychiatric disorders and possibly predict therapeutic effect and outcome.

**Fig. 1:**
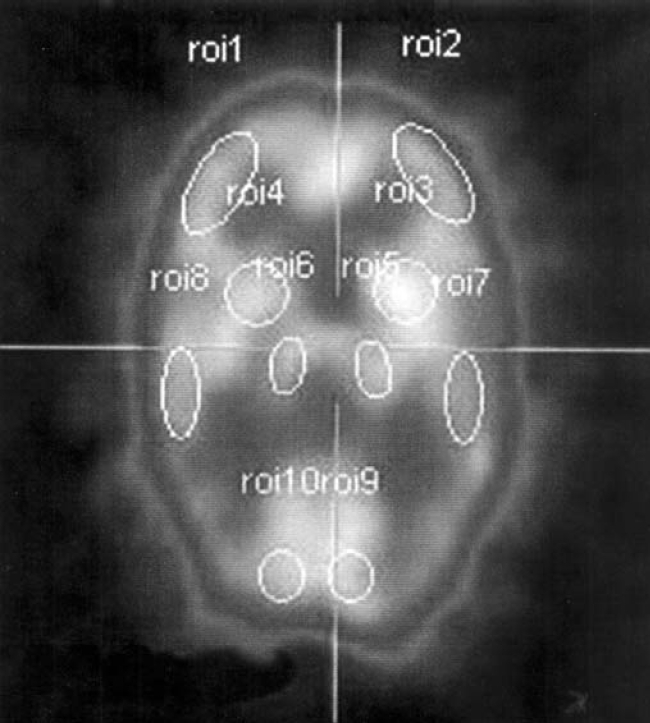
Normal brain SPECT

**Fig. 2:**
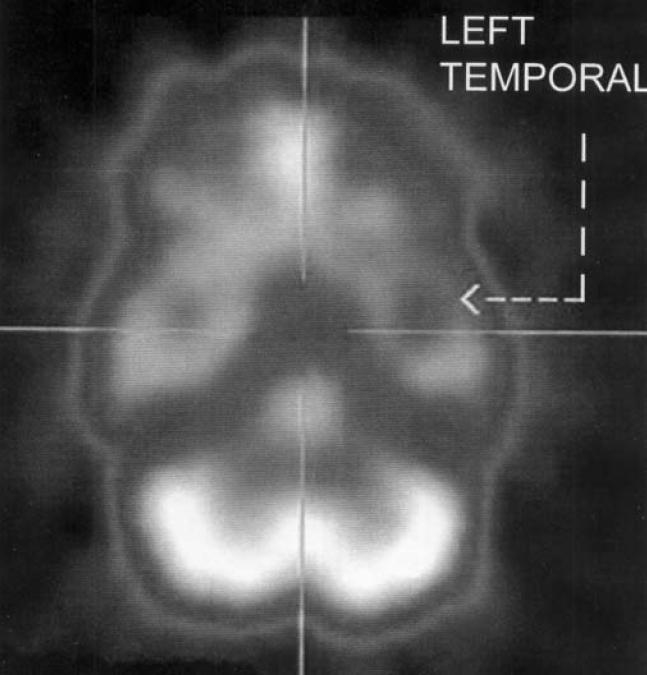
SPECT scan showing reduced perfusion in the left temporal lobe in subject no. 3

**Fig. 3:**
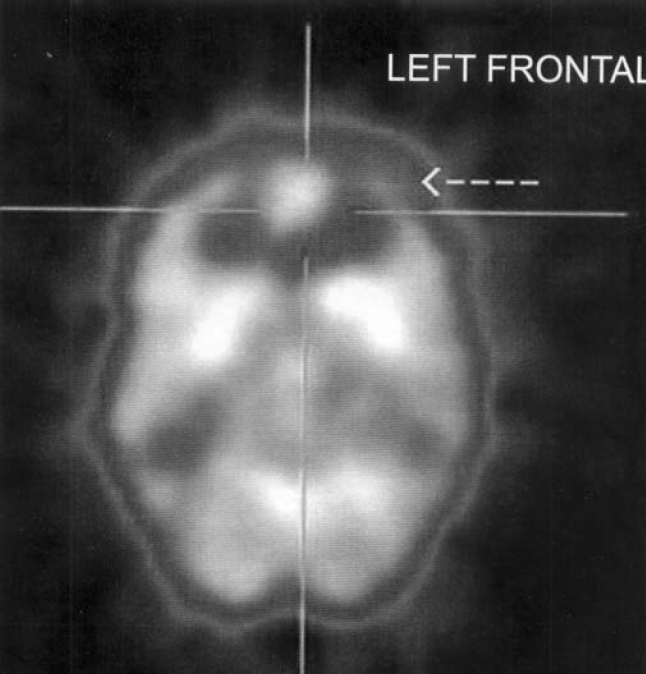
SPECT scan showing reduced perfusion in left frontal lobe in subject no. 5
